# One-domain CD4 Fused to Human Anti-CD16 Antibody Domain Mediates Effective Killing of HIV-1-Infected Cells

**DOI:** 10.1038/s41598-017-07966-3

**Published:** 2017-08-22

**Authors:** Wei Li, Yanling Wu, Desheng Kong, Hongjia Yang, Yanping Wang, Jiping Shao, Yang Feng, Weizao Chen, Liying Ma, Tianlei Ying, Dimiter S. Dimitrov

**Affiliations:** 1 Protein Interactions Section, Cancer and Inflammation Program, Center for Cancer Research, National Cancer Institute, National Institutes of Health, Frederick, Maryland 21702 USA; 2 Key Laboratory of Medical Molecular Virology of Ministries of Education and Health, School of Basic Medical Sciences, Fudan University, Shanghai, 200032 China; 3 State Key Laboratory of Infectious Disease Prevention and Control, National Center for AIDS/STD Control and Prevention (NCAIDS), Chinese Center for Disease Control and Prevention, Beijing, China; 4 Palisades Charter High School, 15777 Bowdoin St, Pacific Palisades, CA 90272 USA; 5 Hainan Medical University, Haikou City, Hainan Province 571199 China

## Abstract

Bispecific killer cells engagers (BiKEs) which can bind to natural killer (NK) cells through the activating receptor CD16A and guide them to cells expressing the HIV-1 envelope glycoprotein (Env) are a promising new weapon for elimination of infected cells and eradication of the virus. Here we report the design, generation and characterization of BiKEs which consist of CD16A binding human antibody domains fused through a flexible linker to an engineered one-domain soluble human CD4. In presence of cells expressing HIV-1 envelope glycoproteins (Envs), these BiKEs activated specifically CD16A-expressing Jurkat T cells, degranulated NK cells, induced cytokine production and killed Env-expressing cells. They also effectively mediated killing of chronically and acutely HIV-1 infected T cells by human peripheral blood mononuclear cells. The presumed ability of these CD4-based BiKEs to bind all HIV-1 isolates, their small size and fully human origin, combined with high efficacy suggest their potential for HIV-1 eradication.

## Introduction

HIV-1 continues to be a major public health problem, and new safer and more effective therapies are needed. Therapeutics approved for clinical use have varying degrees of side effects and none can eradicate the HIV-1. Protein therapeutics are typically cell target-specific and relatively safe^1^. Currently, antibody therapeutics are dominant protein therapeutics with more than 50 monoclonal antibodies (mAbs) approved for clinical use^2^. However, there are no mAbs approved for therapy against any viral diseases. The humanized mAb Synagis is the only one approved by the FDA against a viral disease, however, it is only for prevention and not for therapy^3^.

The identification of novel potent broadly neutralizing antibodies (bnAbs) against HIV-1 during the last several years gave new hopes to the old idea to use antibodies as anti-HIV-1 therapeutics. Attempts to use bnAbs alone or in combination or as components of chimeric antigen receptors (CARs), bispecific T cell engagers (BiTEs) and other bispecific proteins resulted in promising results both *in vitro* and *in vivo* ^4–8^. A major problem with all known bnAbs is that none of them neutralize all HIV-1 isolates which can result in generation of escape mutants.

All HIV-1 isolates (including those few which can enter cells using only a coreceptor) bind to their primary receptor CD4. Recently, we engineered a soluble one domain CD4 (mD1.22) which can bind and neutralize all tested isolates as a component of a bispecific multivalent protein^9^. Thus, we have hypothesized that a bispecific fusion protein containing mD1.22 fused to an antibody arm that binds to CD16A on NK cells, not only would presumably neutralize all HIV-1 isolates but also could guide and activate natural killer (NK) cells to find and kill HIV-1 infected cells expressing its envelope glycoprotein (Env, gp120-gp41) at their surface. The capability of NK cells to kill target cells specifically by using bispecific antibodies to both CD16 and target cells was demonstrated more than 30 years ago^10^. However, it was not until relatively recently when such bispecific molecules called Bispecific Killer cell Engagers (BiKEs) were successfully used against cancer in a clinical trial^11^.

In general, large fusion proteins (150 kDa) do not penetrate the blood-brain barrier (BBB) and their diffusion through the lymphoid tissue where HIV-1 mostly replicates is relatively slow. Decreasing the size of the proteins can dramatically increase their effective diffusion coefficients, e.g., decreasing the size from 150 kDa to about 15 kDa can increase the diffusion through normal solid tissue about 100-fold^12^. Therefore, in an attempt to decrease the size of BiKEs we also used antibody domain binders to CD16A which we recently identified and described^13^. We have hypothesized that BiKEs generated by fusing the one domain CD4 through a flexible linker to an antibody domain binder to CD16A would have relatively small size which could allow them to effectively penetrate tissues and guide the NK cells to kill HIV-1 infected cells.

A major component of a BiKE is the antibody that binds to CD16A. Most (but not all, e.g.,^14^) previously reported antibodies (e.g.,^15^) are from animal origin. The animal antibodies can be humanized and some, e.g., from llama^16^, are similar in sequence to human antibodies; however, the probability for immunogenicity when administered in humans is still on average higher than that for fully human antibodies^17^. Therefore, we used two V_H_ antibody domains (Ads) derived from a human library displayed on phage which bind to CD16A with high affinity, are highly specific and allotype independent^13^.

Here, we show that small BiKEs constructed by fusing these Ads to mD1.22 retain the high affinity, specific binding to both CD16A and gp140. Importantly, in the presence of cells expressing Env, these BiKEs activated specifically the CD16A-expressing Jurkat T cells as well as induced the degranulation of NK cells and resulted in killing of the Env-expressing cells. Furthermore, these BiKEs mediated effective killing of HIV-1 infected T cells by human peripheral blood mononuclear cells (PBMCs). To our knowledge these are the first human domain-based BiKEs and the first against HIV-1 based on CD4; we found only one other report published as an extended abstract about BiKEs against HIV-1 based on an anti-CD16A llama domain and the VRC01 Fab^18^. The possibility that our CD4-based BiKEs can bind all HIV-1 isolates, their small size and fully human origin, combined with their high-affinity and specific, activating interactions with CD16A resulting in lysis of HIV-1 infected cells suggest that they are promising candidate therapeutics and worth of further evaluation in animal models and potentially in humans for therapy of HIV-1 infections with the final goal of its eradication.

## Results

### Design and initial characterization of anti-HIV-1 BiKEs

**Figure 1:**
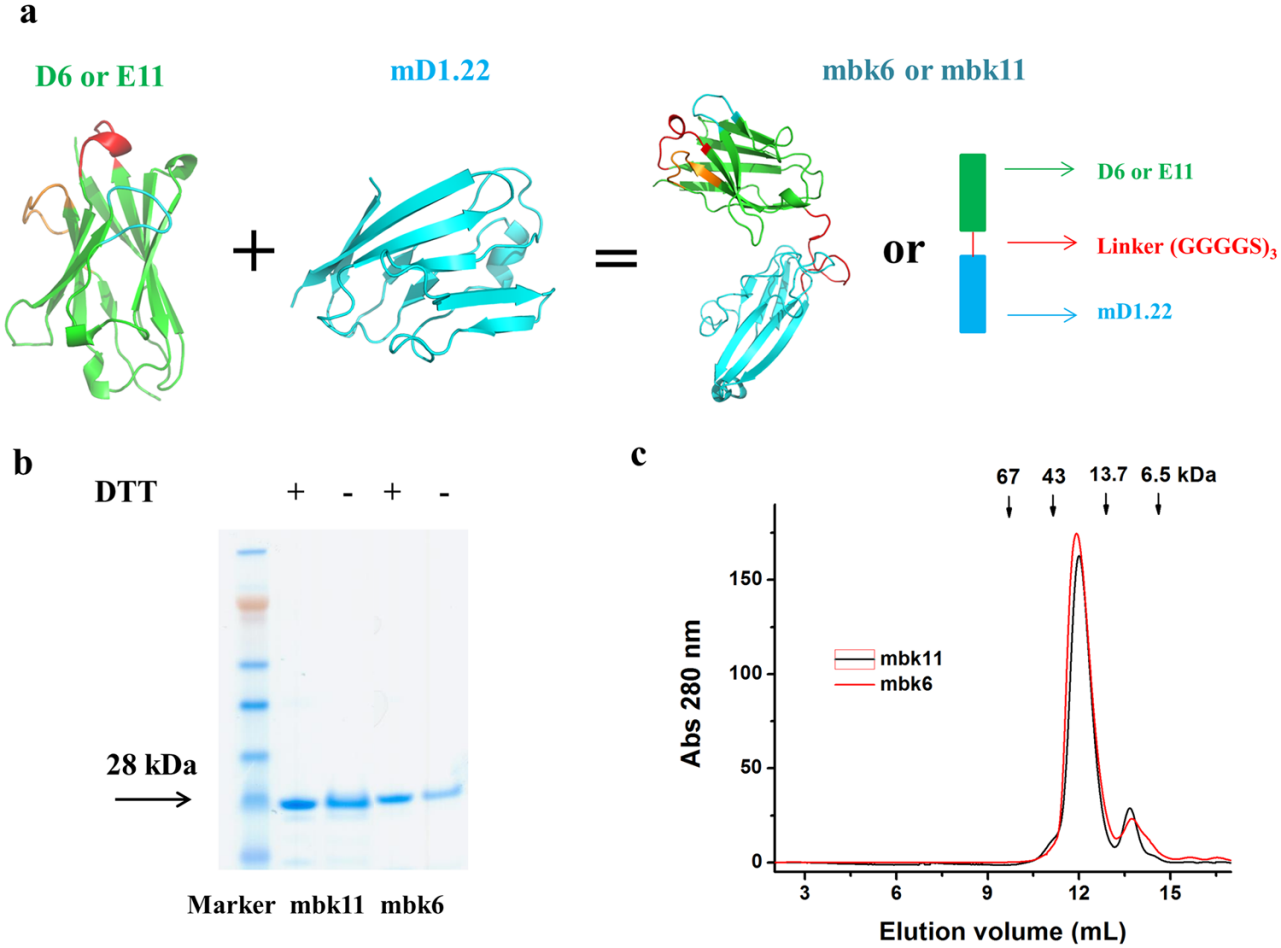
Design and characterization of BiKEs. (**a**) Schematic representation and structural models of the BiKEs. These structures were modeled by Swiss-model^44^, and represented as cartoon models by using PyMOL with green color for the antibody domain targeting CD16A, cyan for mD1.22 and red for the (G_4_S)_3_ linker. The three CDR loops are also highlighted. (**b**) SDS-PAGE in presence (+DTT) or absence (−DTT) of reducing reagent. BiKEs with two immunoglobulin domains have theoretical molecular weight of ~28 kDa. (**c**) Size exclusion chromatography using Superdex 75 10/300 GL. The arrows indicate the peaks of the MW standards in PBS.

We have previously identified antibody domains (Ads), D6 and E11, which bind with high affinity to human CD16A^13^. We fused them through a flexible linker to an engineered one domain soluble CD4 (mD1.22) which binds with high affinity to gp120^9^ **(**Fig. 1a **)**. These BiKEs, designated as mbk6 and mbk11, exhibited single homogeneous bands on both reducing and non-reducing SDS-PAGE gels with molecular weight (MW) corresponding to the calculated ones of approximately 28 kDa **(**Fig. 1b **)**. They were soluble, did not precipitate even at concentrations higher than 30 mg/ml and ran as monomers in size exclusion chromatography **(**Fig. 1c **)**.

### High-affinity binding of BiKEs to recombinant CD16A and gp140

**Figure 2:**
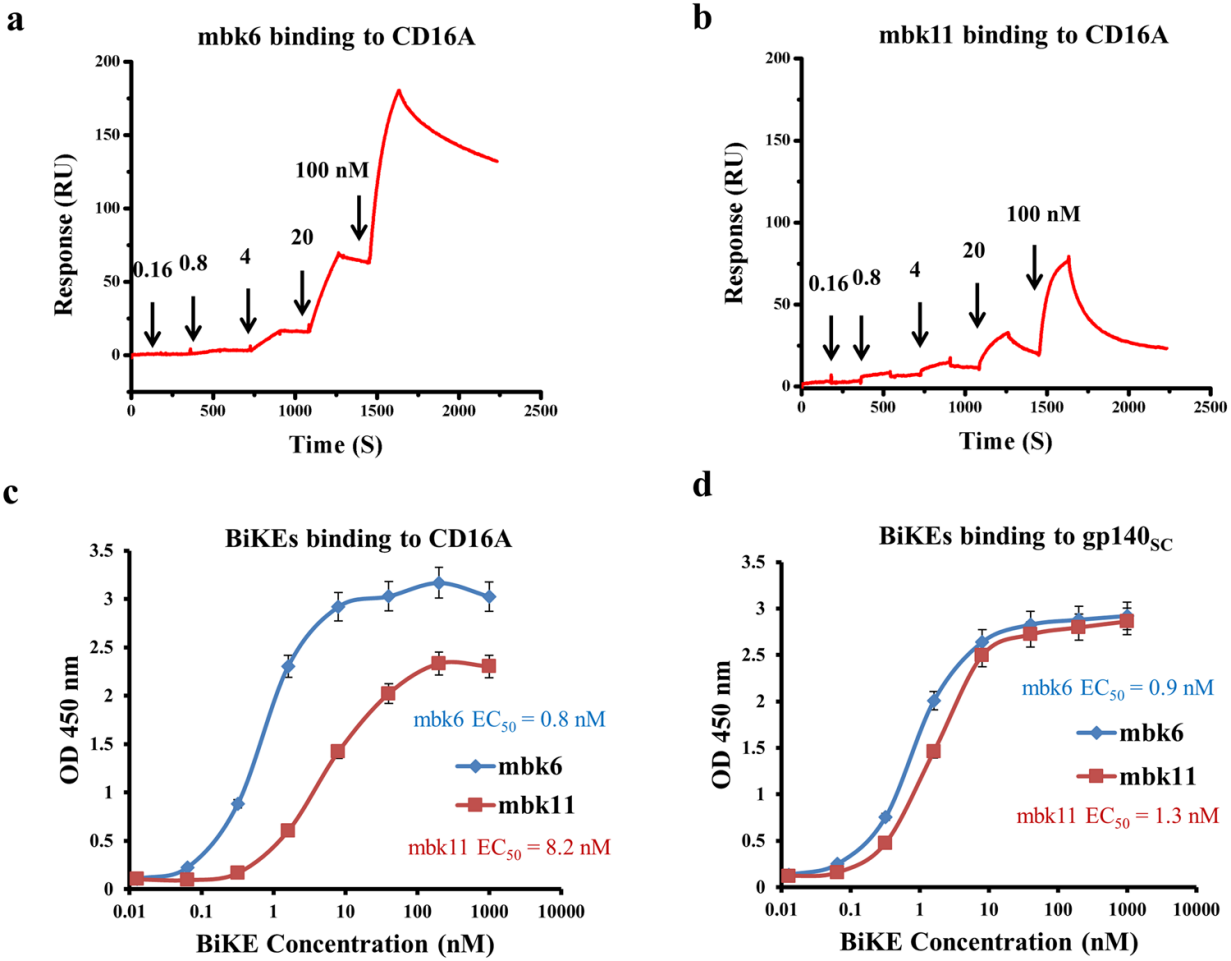
Binding affinity of mbk6 and mbk11 as measured by SPR and ELISA. Panel a and b show the SPR sensorgrams of mbk6 and mbk11, respectively. SPR experiments were performed on a single cycle mode. CD16A was coated onto a CM5 chips and different concentrations of BiKEs was injected. The kinetics constants were obtained by fitting the sensorgrams. Panel c and d show binding to CD16A and gp140sc, respectively, measured by ELISA. 50 ng/well of CD16A-mFc and gp140sc were coated onto costar half area, high binding, polystyrene 96 wells plate and incubated with serially diluted BiKEs for 2 hours at room temperature, after extensive washing by PBS + 0.05% Tween-20, bound BiKEs were detected by using HRP conjugated anti-FALG antibody. Experiments were performed in triplicate and the error bars denote ± SD, n = 3.

We have previously showed that the antibody domain D6 possesses 10-20 folds higher affinity to CD16A than E11^13^. The affinity of mbk6 containing D6 to CD16A was also higher than that of mbk11 as measured by Biacore **(**Fig. 2a,b **)**. The association rate constant of mbk6 was lower than that of mbk11 (1.1 × 10^5^ *vs*. 4.7 × 10^5^ M·s^−1^, respectively) but the dissociation rate constant was much lower (1.2 × 10^−4^ *vs*. 1.0 × 10^−2^ s^−1^, respectively), resulting in the lower equilibrium dissociation constant, *K*_d_, and higher affinity of mbk6 compared to that of mbk11 (*K*_d_ *= *1.1 nM *vs*. 21 nM, for mbk6 and mbk11, respectively). Similar high affinity binding was also measured by using ELISA although the EC_50_s were somewhat lower (0.8 *vs*. 8.2 nM) **(**Fig. 2c **)**. These two BiKEs also retained their high affinity (EC_50_, ~1 nM) binding to gp140sc through its mD1.22 arm as measured by ELISA **(**Fig. 2d **)**. As expected, mbk6 and mbk11 have similar binding affinity to gp140sc. Taken together, these data demonstrate a bispecific, high-affinity binding of the new BiKEs to both CD16A and gp140.

### Specific binding of mbk6 and mbk11 to cell surface-associated CD16A and gp160

**Figure 3:**
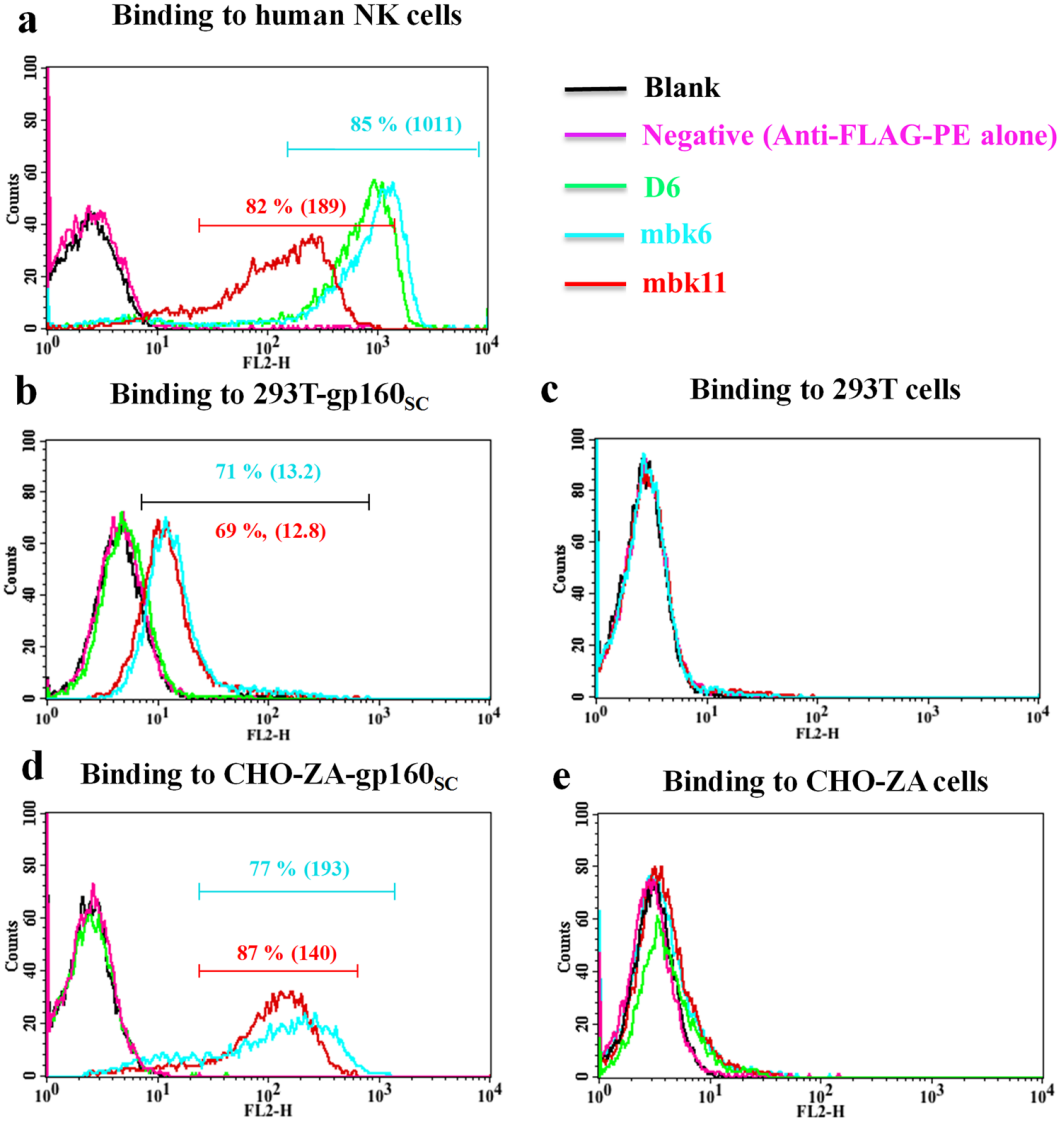
Binding of mbk6, mbk11 and D6 (at 10 nM) to human NK cells, 293 T cells with or without surface-associated gp160, and gp160+/− CHO-ZA cells. The cells were incubated with BiKEs or D6 followed by detection with PE conjugated anti-FLAG antibody. All experiments were performed in triplicate, n = 3. (**a**) BiKEs binding to human NK cells expressing CD16A. (**b**) Binding to 293T-gp160_SC_ cells. **(c)** Binding to 293 T cells. (**d**) Binding to CHO-ZA-gp160_SC_ cells. (**e**) Binding to CHO-ZA cells.

We next evaluated whether these two BiKEs bind specifically to CD16A and gp160 expressing cells **(**Fig. 3 **)**. Both mbk6 and mbk11 bound strongly to the purified NK cells at a low concentration (10 nM) as measured by flow cytometry **(**Fig. 3a **)**. As expected mbk6 bound to NK cells more efficiently than mbk11 and comparably to the Ad D6. Both BiKEs bound comparably to 293 T cells and CHO-ZA cells permanently expressing gp160 (gp120-gp41) (Fig. 3b,d **)** but not to un-transfected 293 T cells and CHO-ZA cells (Fig. 3c,e **)**. Interestingly, the fluorescence shift for BiKEs binding to CHO-ZA-gp160sc cells was substantially larger than binding to 293T-gp160sc cells, which is consistent with binding of other two Env binders, b12^19^ and LSEVh-LS-F^20^, to these two cell lines **(**Supplemental Fig. 1 **)** and indicated that CHO-ZA cells expressed higher level of surface-associated gp160sc than 293 T cells did after transfection. These results suggest that mbk6 and mbk11 bind specifically to both cell surface associated CD16A and gp160.

### Specific activation of Jurkat T–CD16A cells mediated by BiKEs

**Figure 4:**
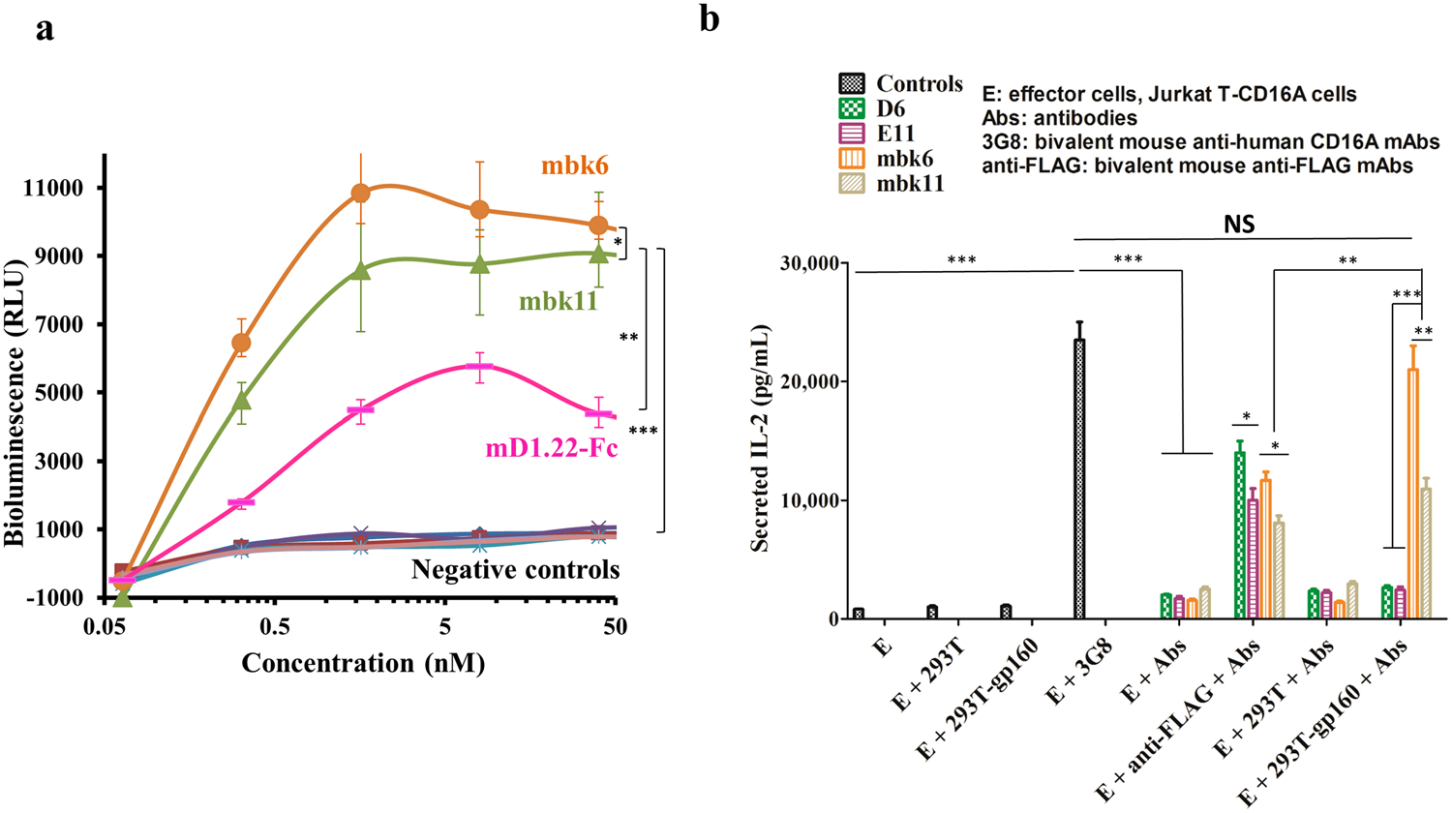
Activation and IL-2 release by Jurkat T-CD16A cells after CD16A engagement by anti-CD16A antibodies or BiKEs. Activation was measured by monitoring luciferase activity. IL-2 secretion was tested by sandwich ELISA by using the Duoset Human IL-2 kit from R&D Systems. (**a**) Luciferase activity (Unit, RLU; RLU stands for relative light unit) of Jurkat T-CD16A cells after incubation with BiKEs or mD1.22-Fc. Experiments were performed in triplicate.  Represents Jurkat T-CD16A cells incubated with mbk6 and 293T-gp160_SC_ cells (Jurkat T-CD16A + mbk6 + 293T-gp160_SC_, simply denoted as mbk6 in the figure); , Jurkat T-CD16A + mbk11 + 293T-gp160_SC_ (denoted as mbk11); , Jurkat T-CD16A + mD1.22-Fc + 293T-gp160_SC_ (simply put as mD1.22-Fc); other groups are negative controls, in which Jurkat T-CD16A cells was either incubated with BiKEs in the absence of target cells, or incubated with BiKEs in the presence of gp160_SC_ negative 293 T cells. , Jurkat T-CD16A + mbk6; , Jurkat T-CD16A + mbk6 + 293 T; , Jurkat T-CD16A + mbk11;  Jurkat T-CD16A + mbk11 + 293 T; , Jurkat T-CD16A + mD1.22-Fc; , Jurkat T-CD16A + mD1.22-Fc + 293 T. (**b**) Secretion of IL-2 by Jurkat T-CD16A cells activated by BiKEs at 20 nM. The results shown are from three independent experiments. Statistical tests were performed using GraphPad Prism5. Significant differences when comparing two groups were determined by two-way ANOVA (panel a) and Student’s *t* test (panel b). A two-tailed *p* value < 0.05 was considered significant. *p < 0.05. **p < 0.01. ***p < 0.001. NS: not significant.

We assessed whether the BiKEs, after engaging cell surface CD16A, could mediate functional activation of CD16A expressing Jurkat T cells (Jurkat T-CD16A cells) by monitoring their activation and cytokine (IL-2) secretion. In Jurkat T-CD16A cells, engagement of CD16A initiates activation signals through the CD3-ζ chain, ultimately leading to de-phosphorylation of NTAFp (Nuclear factor of activated T-cells) and expression of luciferase (Promega, ADCC Reporter Bioassay). Thus, the activation signals could be monitored by testing luciferase activity. To compare the ADCC capacities of BiKEs and IgG1-Fc, we also measured the ADCC activation signals mediated by mD1.22-Fc, an Fc fusion protein with bivalent mD1.22. BiKEs and mD1.22-Fc can specifically activate Jurkat T-CD16A cells in the presence of 293T-gp160_SC_ cells **(**Fig. 4a **)**. The incubation of BiKEs with Jurkat T-CD16A cells in the absence of target cells or in the presence of 293 T cells non-expressing gp160 could not induce luciferase signals, demonstrating the high specificities of these BiKEs. Importantly, they can activate Jurkat T-CD16A cells at low concentrations (50% RLU at 0.2 nM). In this case the activity of mbk6 was higher than that of mbk11 and mD1.22-Fc indicating a correlation of binding affinity of BiKEs to CD16A (mD1.22-Fc < mbk11 < mbk6) with their functional activity.

We also measured cytokine (IL-2) secretion of Jurkat T-CD16A cells bound to BiKEs after cross-linking, which was achieved by either bivalent mouse anti-FLAG antibody or by 293T-gp160_SC_ cells. Co-incubation of Jurkat T-CD16A cells with BiKEs opsonized 293T-gp160_SC_ cells resulted in release of IL-2 **(**Fig. 4b **)**, which is consistent with the results obtained by using a positive control, bivalent 3G8. By contrast, the BiKEs alone or incubated with 293 T cells, or 293T-gp160_SC_ alone did not induce IL-2 secretion, demonstrating the high specificity of these BiKEs. The higher affinity mbk6 induced more IL-2 than mbk11 (*p* = 0.022) but less than 3G8, which again indicates that there could be correlation between affinity/avidity and cytokine release activity. However, when T cells were stimulated by 293T-gp160_SC_ cells, mbk6 was almost as effective as 3G8 suggesting a role for avidity effects. These results demonstrate that both BiKEs could specifically engage CD16A on the effector cell surface, and activate signals leading to cytokine release.

### NK cells degranulation mediated by BiKEs

**Figure 5:**
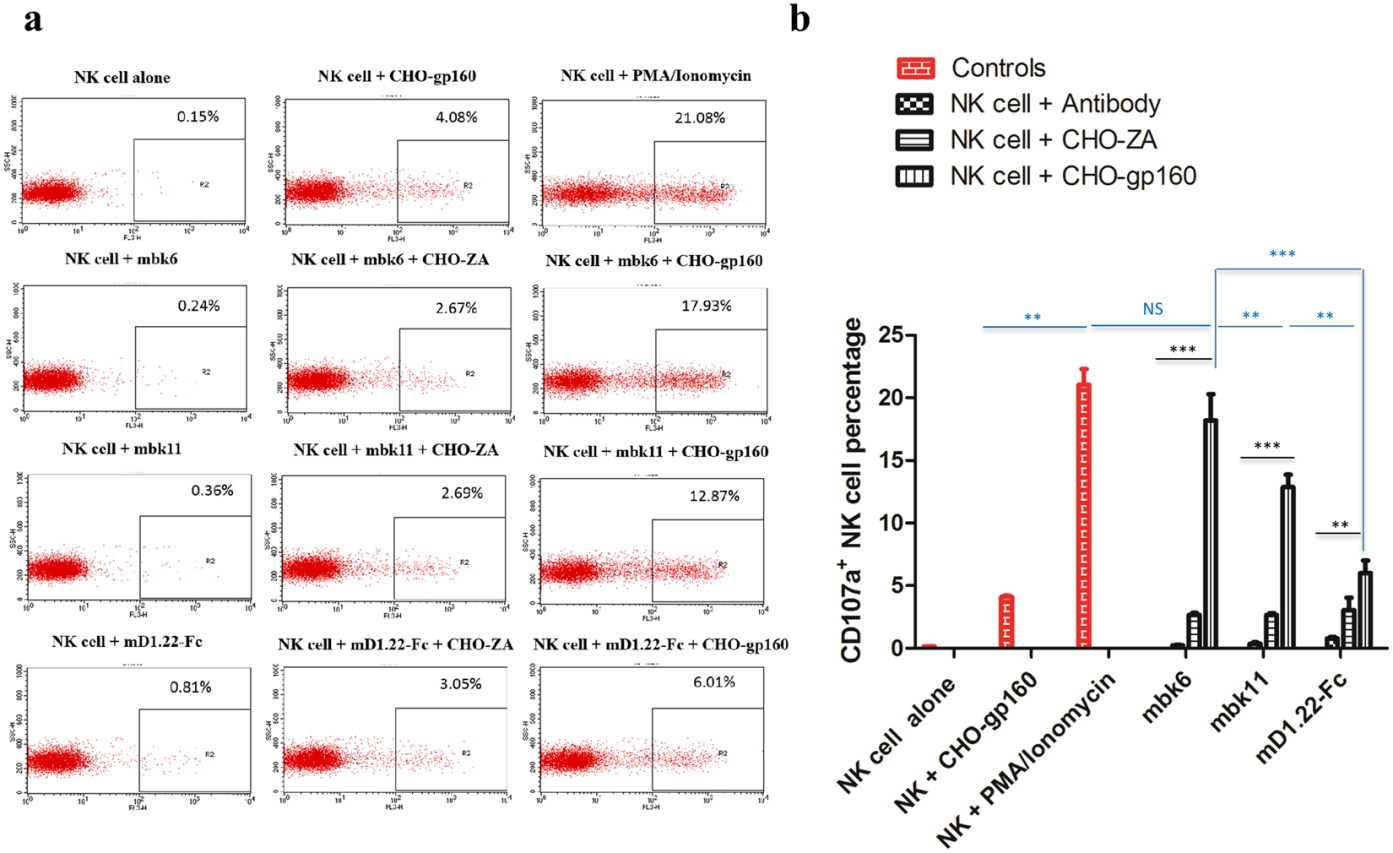
NK cells degranulation mediated by BiKEs or mD1.22-Fc. Degranulation was evaluated by the NK cell surface CD107a staining using the PE-Cy^TM^5 mouse anti-human CD107a antibody (**a**) CD107a expression on NK cells surface. NK cells were incubated with 20 nM of BiKEs or mD1.22-Fc in the absence or presence of CHO-ZA-gp160_SC_. The data were analyzed by the CellQuest Pro software. (**b**) Percentage of CD107a positive NK cells. The red color in the column figure denotes the controls without BiKEs and the black color accounts for the BiKEs incubation with NK cells alone, or combined with CHO-ZA cells or CHO-ZA-gp160_SC_ cells. The results are from three independent experiments. Error bars were calculated and statistical significance was tested by using GraphPad Prism5. Significant differences among inter-groups (blue color) and intra-group (black color) were determined by two-way ANOVA and Student’s t test, respectively. A *p* value < 0.05 was considered significant. *p < 0.05. **p < 0.01. ***p < 0.001. NS: not significant.

Next we measured BiKE-mediated degranulation of NK cells in the presence of CHO-ZA-gp160_SC_ cells. NK degranulation was evaluated by monitoring CD107a and IFNγ expression levels after incubation with BiKE-opsonized target cells. To assess the specificity of these BiKEs, we used three negative controls: NK cells incubation with BiKEs in the absence of target cells, in the presence of HIV-1 Env negative cells or NK cells incubation with target cells in the absence of BiKEs. NK cells stimulated with PMA/ionomycin were used as a positive control. The CD107a staining results **(**Fig. 5a **)** clearly showed that both mbk6 and mbk11 induced significant NK cell degranulation compared to the negative controls **(**Fig. 5a,b **)**.

Mbk6 degranulated NK cells more effectively than mbk11, and both BiKEs were more effective than mD1.22-Fc, which correlates with Jurkat T-CD16A cells activation and indicates that the affinity to CD16A is important for the extent of activation of CD16A-expressing effector cells. It should be also noted that the CHO-ZA-gp160_SC_ cells and CHO-ZA cells in the absence of BiKEs also could induce relatively weak NK cells degranulation, which may result from killer immunoglobulin-like receptors (KIRs) and natural cytotoxicity receptors (NCRs) recognition of Env or other ligands on target cells^21^. This is independent on CD16A mediated NK cells engagement. Induced expression of intracellular IFNγ was also observed although the level was not as high as the CD107a expression **(**Supplemental Fig. 2 **)**. This is consistent with previous observation that CD107a is a more comprehensive and sensitive marker of NK cells activation than cytokine secretion^22^. It should be noted that our CD107a and IFN-γ levels are lower than those induced by the VRCO1 based BiKEs^18^, which may result from the different experimental conditions such as different Env expression levels or NK cells donors or incubation time. These results demonstrate that BiKEs can specifically engage NK cells in the presence of gp160-expressing target cells.

### Specific killing of Env-expressing and HIV-1-infected cells mediated by mbk6 and mbk11

**Figure 6:**
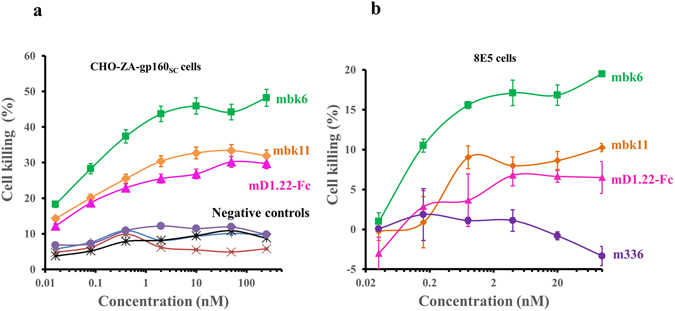
Specific BiKE-mediated killing. (**a**) Killing of CHO-ZA-gp160_SC_ cells in presence of NK cells. Target cells were pre-labelled by PHK26 and incubated with serially diluted BiKEs or mD1.22-Fc followed by adding NK cells. The dead cells were measured by PI staining before evaluation by FACS analysis. Experiments were performed in triplicate and the error bars denote ± SD, n = 3.  Represents NK cells incubated with mbk6 and CHO-ZA-gp160_SC_ cells (NK cells + mbk6 + CHO-ZA-gp160_SC_, denoted as mbk6 in the figure); , NK cells + mbk11 + CHO-ZA-gp160_SC_ (denoted as mbk11); , NK cells + mD1.22-Fc + CHO-ZA-gp160_SC_ (denoted as mD1.22-Fc); other groups are **negative controls**, in which NK cells incubation with CHO-ZA-gp160_SC_ cells in the absence of BiKEs, or NK cells incubated gp160 negative CHO-ZA cells in the presence of BiKEs or mD1.22-Fc. , NK cells + mD1.22-Fc + CHO-ZA; , NK cells + CHO-ZA-gp160_SC_; , NK cells + mbk6 + CHO-ZA; , NK cells + mbk11 + CHO-ZA. (**b**) Killing of chronically HIV-1 infected cells (8E5) by human PBMCs mediated by BiKEs. Experiments were performed by co-incubation of PBMCs with BiKE opsonized 8E5 cells and the assay was developed by detecting LDH activity using Promega CytoTox-ONE™ Homogeneous Membrane Integrity kit. The anti-MERS-CoV mAb, IgG1 m336 was used as a negative control. Experiments were performed in duplicate and the error bars denote ± SD, n = 2.

We used three different models to assess the killing activity of the new BiKEs. The first one is based on the CHO-ZA cell line which was transfected to permanently express gp160_SC_. In this case cell killing was measured by a flow cytometry-based method where target cells were discriminated from effector cells by pre-labelling with the fluorescent dye PKH26. After incubation with BiKEs and effector cells, the dead target cells were further differentiated by PI staining. Both BiKEs and mD1.22-Fc specifically mediated cell killing by NK cells only in the presence of CHO-ZA-gp160_SC_ cells **(**Fig. 6a **)**. Mbk6 was most effective with 50% of the maximal killing at a very low concentration of < 100 pM. The cell killing efficacy of mbk11 was lower than that of mbk6. The mD1.22-Fc with the lowest affinity to CD16A exhibited the lowest cell killing efficacy, which correlates with its capacity to induce NK cell degranulation. The killing activity of mbk6 was significantly reduced in the presence of 10 nM D6 indicating specific killing of target cells by mbk6 **(**Supplemental Fig. 3).

We evaluated BiKEs mediated killing of 8E5 cells by using a different assay based on measuring membrane integrity. 8E5 cells are chronically HIV-1_LAV_-infected cells which stably express most HIV-1 structural proteins including the Env and are frequently used for modeling of chronic HIV-1 infections. In this case again mbk6 was most effective in mediating 8E5 cell killing by PBMCs **(**Fig. 6b **)**. Both BiKEs were more effective than mD1.22-Fc, which correlates with their affinity and activity in all assays described above.

In another set of experiments we used acutely infected T cells (CEM.NK^R^CCR5^+^ cells infected by the HIV-1_NL4-3_) as target cells. The ADCC assay was performed four-days post-infection when the infected CEM cells expressed significant amount of Env but death due to the HIV-1 infection *per se* was relatively low. ADCC assays showed that at low concentrations of BiKEs and mD1.22-Fc (0.8 nM), both BiKEs as well as mD1.22-Fc mediated specific killing of the infected CEM cells by PBMCs **(**Supplemental Fig. 4 **)**. The BiKEs were more effective than mD1.22-Fc. We also used a primary HIV-1 isolate (2016GXEU02) to infect CEM.NK^R^CCR5^+^ cells. We detected BiKEs mediated HIV-1 killing by monitoring luciferase activity of target cells. We observed higher killing activity (up to 70%) (data not shown). These results suggest that these BiKEs are promising candidates for further evaluation in animal models and eventually in humans.

## Discussion

The important role of NK cell mediated ADCC in HIV-1 infections has been well documented in the past decades^23–25^. ADCC in HIV-1 patients was mediated by endogenous IgGs or by exogenous therapeutic mAbs (IgG1) through their Fc binding to the activating receptor CD16A on the surface of the NK cells^26^. Here we demonstrated that the ADCC function of the NK cells could be alternatively induced by BiKEs consisting of a CD16A binding antibody domain and a soluble one-domain CD4 (mD1.22). To our knowledge, mbk6 and mbk11 are among the first reported BiKEs against HIV-1 infection although we are aware that there are ongoing studies. After this study was completed an extended abstract was recently published^18^.

Using BiKEs against HIV-1 is a promising new approach. NK cells as effector cells are relatively refractory to HIV-1 infection. Although it was reported that CD56^high^ NK cells expressing CD4, CCR5 and CXCR4 could be infected by HIV-1^27^, the cytolytic CD56^dim^CD16^high^ NK cells rarely express CCR5 and CXCR4^28^. Besides, during HIV-1 infection and progression, the phenotype of NK cells can shift from cytokine secretion CD56^high^ population to CD16^high^ phenotype with expanded cytotoxicity along with diminished CXCR4 and CCR5 expression^28^, indicating that NK cells are effective effector cells against HIV-1. In addition, NK mediated cell killing mediated by CD16A doesn’t rely on KIR/HLA-I matching or is not subjected to inhibition by other NK cells inhibitory receptors for which HIV-1 has evolved strategies to escape^24^. Thus, recruiting NK cells by targeting CD16A could be an effective novel strategy against HIV-1 infection.

A major unique feature of our BiKEs is that binding to the HIV-1-infected cells is mediated by our one-domain CD4 which binds to all HIV-1 isolates tested^9^. Therefore, one could hypothesize that our BiKE construct could be presumably able to kill cells infected with all isolates, and escape of resistant virus may be difficult although future experiments are needed to prove this statement. Furthermore, one can envision that mD1.22 in the BiKEs could also act as an HIV-1 entry inhibitor independent of its effect on NK cells, which could lead to synergistic effects.

Another unique feature of our BiKEs is that they are based on fully human molecules. The CD16A engagement moiety in our BiKEs is based on antibody domains, which were developed from a human V_H_ library by using phage display. These antibody domains have high affinity (1–10 nM), specificity and allotype independent binding to CD16A^13^, which are highly desirable properties for efficacy *in vivo*. Thus, mbk6 and mbk11 could potentially bypass the hurdles encountered by IgG-Fc mediated ADCC, such as competition by endogenous IgG^29^ and limitations due to the CD16A 158 V/F polymorphism^30^ as well as non-specific engagement of other FcλRs^31^. Our antibody domains are also devoid of N-glycosylation, i.e., they do not contain glycans (as Fc does) which could complicate IgG-Fc mediated ADCC by different glycoforms^32^.

Mbk6 and mbk11 were constructed by linking two single fully human domains via three repeats of a poly-peptide, GGGGS. Unlike the typical BiKEs consisting of two tandem single chain variable fragments (scFv), which usually encounter undesirable expression and folding issues^33^, our BiKEs only contain two immunoglobulin domains with total molecular weight of ~ 28 kD **(**Fig. 1b **)**. This simple format renders our BiKEs with high expression in *E. coli* (50 mg/L) and homogenous folding in solution **(**Fig. 1c **)**. To our knowledge, these are the first BiKEs reported which are entirely based on domains and therefore are of relatively very small size. The small size is important because their penetration in normal tissues including lymphoid tissue where HIV-1 mostly replicates could be significantly higher than antibody fragments and antibody fusion proteins. For example, a previous study has shown that a full size antibody (IgG1) of MW about 150 kDa has an effective diffusion coefficient about 100-fold lower in solid tissues than molecules 10-fold smaller^12^. Therefore, the effect of size could be significant. Our BiKEs are also fully human and may not exhibit immunogenicity if administered to humans. However, only further experiments in animal models of HIV-1 infection and ultimately human clinical trials will show whether they are immunogenic or not, and if immunogenic how immunogenic they are. Previously, a llama antibody domain that binds with high affinity to CD16A was identified^16^ and used for generation of two BiKEs targeting carcinoembryonic antigen (CEA)^34^ and Her2^35^. These BiKEs were successful in CD16A mediated lysis of target cells thus demonstrating for the first time that antibody domains are functional as components of BiKEs. Here, we present evidence confirming such statement and further expanding it with the use of fully human domains fused to another human protein domain (mD1.22).

The BiKEs described here bound specifically and with high affinity to both CD16A and gp140 as recombinant proteins and as cell surface associated proteins **(**Figs 2 and 3 **)**. The binding is functional, as demonstrated by multiple experimental evidences in this study. Mbk6 and mbk11 activated Jurkat T-CD16A cells by dephosphorylating NFATp followed by luciferase expression and cytokine IL-2 secretion. These BiKEs could also induce NK cells degranulation after encountering target cells and the activated NK cells could kill target cells expressing the HIV-1 Env (CHO-ZA-gp160_SC_). Importantly, our small BiKEs could effectively engage PBMCs to kill chronically HIV-1 infected cells (8E5 cells) constitutively expressing the Env as well as acutely HIV-1 infected CEM cells by a subtype B strain, HIV-1_NL4-3_ and a recent primary isolate 2016GXEU02. These results are very promising although only further experiments in animal models of HIV-1 infection and ultimately human clinical trials will show whether they are highly effective *in vivo*.

It should be noted that the efficacy of BiKEs engagement of effector cells is exceptionally high. BiKEs stimulated Jurkat T-CD16A cells and induced killing of CHO-ZA-gp160_SC_ and 8E5 cells with IC_50_s as low as ~ 100 pM **(**Figs 4 and 6 **)**. Interestingly, in these assays, mbk6 could engage effector cells more efficiently than mbk11, and mbk11 was more effective than mD1.22-Fc, which correlates with their CD16A binding affinity: mbk6 (~1 nM) > mbk11 (~21 nM) > Fc (0.1–1 μM). These results indicate that binding affinity of mbk6, mbk11 and mD1.22-Fc to CD16A correlates with their functional efficacy, given their identical epitopes on gp120 (CD4 binding site) and similar epitopes on CD16A^13^. Therefore, one could speculate that our high affinity BiKEs could function more effectively than HIV-1 bnAbs with CD4 binding site epitopes although we did not compare side by side the ADCC efficacy of BiKEs and bnAbs in this study.

The high affinity to CD16A for these BiKEs could be also important in chronically HIV-1 infected patients where constant activation may exhaust NK cells^36^, leading to decreased expression of CD16A due to cleavage by the matrix-metalloprotease ADAM17^37^; another example of the numerous ways HIV-1 uses to escape immune surveillance. The high affinity to CD16A of BiKEs could result in binding to NK cells even when the CD16A expression level is low. Another consideration is that NK cells in the solid lymphoid organs and mucosae, where HIV-1 mostly replicates, are mainly CD56^pos^ phenotype, rather than the cytotoxic CD16^pos^ due to the decreased expression of the tissue-homing receptors on CD16^pos^ NK cells during the HIV-1 progression^28, 38^. The high affinity to CD16A could maximize the engagement of the low CD16^pos^ cell number in solid lymphoid tissues to kill HIV-1-infected cells. It can be also important in cases when the Env is expressed at low surface concentrations especially in chronically and latently infected cells^39^. Therefore, the high binding affinity of our BiKEs to both CD16A and gp120 could be important for their *in vivo* efficacy.

The high affinity, however, could lead to specificity issues. Thus, our cell killing assays incorporated various controls to determine possible non-specificity of these BiKEs. All results, including activation and cytokine secretion by Jurkat T-CD16A cells, NK cell degranulation and killing of Env-expressing cells or HIV-1 infected cells, consistently were highly dependent on the presence of both BiKEs and target cells. BiKEs alone could neither activate Jurkat T-CD16A, nor induce degranulation of NK cells. Co-incubation of target cells with effector cells in the absence of BiKEs or co-incubation of Env-negative control cells with effector cells in the presence of BiKEs could not lead to the target cell killing. Although in the NK cell degranulation assays, co-incubation of NK cells with CHO-ZA-gp160_SC_ cells in the absence of BiKEs did induce very low expression of CD107A, it may result from the direct engagement of other receptors on NK cell surface such as KIRs and NCRs^21, 24^, which is independent of CD16A and BiKEs. Of note is that we have previously shown that one-domain CD4 (mD1.22) doesn’t bind MHC class II molecules^9^. Thus, our data suggest that the activity of these BiKEs is highly specific.

The new BiKEs are not only highly functional in killing HIV-1 infected cells and the only reported fully human BiKEs based on protein domains that exhibit highly specific, high-affinity binding but they also possess good drugability properties including relatively high level of expression. Therefore, alone or in combination with other HIV-1 inhibitors they could be useful in the development of novel safe therapeutics against HIV-1 and potentially as tools for its eradication.

## Methods

### Proteins, plasmids, antibodies, cells and virus

Recombinant CD16A-mFc, D6, gp140sc, mD1.22-Fc, m336 and LSEVh-LS-F were produced in our group as previously described^13, 20, 40, 41^. The CD16A binding antibody domain D6 was expressed in HB2151 bacteria and purified by Ni-NTA resin (Qiagen, Valencia, CA). CD16A-mFc was subcloned into pSecTag B vector and expressed in 293 FreeStyle^TM^ cells, and purified by using Protein A sepharose resin (Invitrogen). Gp140sc was codon optimized from the Env sequences of a clade-B HIV-1 isolate, and synthesized, subcloned into pSecTag B, expressed in CHO-ZA cells and purified by Ni-NTA. MD1.22-Fc is an Fc fusion protein with mD1.22 expressed in 293 FreeStyle^TM^ cells and purified by Protein A resin^9^. M336 used as the negative control in HIV-1 infected cells killing experiment is a human monoclonal antibody IgG1 targeting the MERS-CoV^41^. M336 cloned in pDR12 vector was expressed in CHO-ZA cells and purified by Protein A resin. PComb3X and pDR12 vector were kindly provided by Dennis Burton (Scripps Research Institute, La Jolla, CA, USA). The pSecTag B vector was purchased from Invitrogen. The following antibodies were purchased: mouse anti-CD16A IgG1, 3G8 (Abcam, Cambridge, MA, USA); phycoerythrin (PE)-conjugate mouse anti-FLAG, phycoerythrin (PE)-conjugate mouse anti-CD16A (Mouse IgMκ, clone VEP13) (Miltenyi, Bergisch Gladbach, Germany); HRP-conjugated mouse anti-FLAG tag (Sigma-Aldrich). CHO-ZA cell is a suspension derivative of CHO-K1 cells adapted to non-serum medium. CHO-ZA gp160_SC_ cell is a stable cell line developed in our laboratory (DSD) which expresses gp160; it is cultured in optiCHO medium (Invitrogen) containing 31.5 µg/ml zeocin and 2 mM glutamine. Similarly, 293 T cells expressing gp140_SC_ were maintained in DMEM complete medium (Invitrogen) containing 10% FBS (Invitrogen) and 60 µg/ml zeocin. The following cell lines were purchased: 293 T, CHO-K1 and 8E5/CEM cells constitutively expressing HIV-1_LAV_ Env (ATCC); 293 FreeStyle^TM^ (Invitrogen) and CD16A expressing Jurkat T cells (Promega). CEM.NK^R^CCR5-Luc cells were provided by Dr. Alexandra Trkola to the AIDS Research and Reference Reagent Program (National Institute of Allergy and Infectious Diseases, Bethesda, MD). The HIV-1 primary isolate was isolated from a Chinese HIV-1 positive subject (2016GXEU02). Human blood was obtained from healthy donors with an approved human subject agreement. Collections of blood from donors were approved by National Cancer Institute-Frederick Research Donor Program.

### Generation and purification of BiKEs

The BiKE6 and BiKE11 gene were obtained by fusing D6 and E11 DNA to mD1.22 DNA through overlapping extension PCR using the following primers: Vh-mD1.22F1: 5-ACGCGGCCCAGCCGGCCGAGGTGCAGCTGGTGGAGTCTGGG-3 (sense); Vh-mD1.22R1: 5-TCCAGACCCTCCACCGCCACTTGAGGAGACGGTGAC-3 (anti-sense); Vh-mD1.22F2:5-GGCGGTGGAGGGTCTGGAGGGGGCGGGAGCGGAGGCGGTG GCTCGAAGAAGGTGGTGTACGGCAAGAAG-3(sense); Vh-mD1.22R2:5-ATAGGCCGGCCTGGCCGCCTACCACT ACCAGCTGCACCTC-3 (anti-sense). The Sfi I digested BiKE gene were then subcloned into Sfi I linearized pComb3X vector containing an in-frame C terminal 6 × His tag followed by a FLAG tag (DYKDDDDK). The recombinant BiKE plasmids were expressed in HB2151 cells and were purified by one-step Ni-NTA chromatography. The purity was checked by SDS-PAGE under both reducing and non-reducing conditions **(**Fig. 1b **)**, and the homogeneity was confirmed by Superdex 75 10/300 GL chromatography (GE Healthcare, Cat. No. 17-5174-01) **(**Fig. 1c **)**. The column was calibrated with protein molecular mass standards of 1.355 kDa B12, 6.5 kDa aprotinin, 13.7 kDa ribonuclease, 43 kDa ovalbumin and 67 kDa BSA. Purified BiKEs in PBS were loaded into a pre-equilibrated column and eluted with PBS at 0.5 ml/min. Protein concentration was measured spectrophotometrically (NanoVue, GE Healthcare). The protein was stored at −80 °C until use. We noticed that storage could change its properties but not to significant degree to affect conclusions.

### ELISA

96-well plates (Costar) were coated with CD16A-mFc or gp140sc at 50 ng/well in phosphate-buffered saline (PBS) overnight at 4 °C followed by blocking with PBS containing 3% non-fat milk (MPBS) for 1 h at room temperature. Then the plates were incubated with serially diluted BiKEs (5 folds with a beginning concentration of 1 μM) for 2 h at room temperature. After extensive washing with PBS containing 0.05% Tween-20, the bound BiKEs were detected by HRP-conjugated mouse anti-FLAG tag and the signals were recorded by monitoring the optical absorbance at 450 nm after adding the substrate TMB (3,3′,5,5′-Tetramethylbenzidine, (Sigma-Aldrich). The half-maximal binding (50% effective ELISA Concentration, EC_50_,) was designated as the concentration at which optical density (OD) is 50% of its maximal value.

### Surface plasmon resonance (SPR)

The kinetics of binding of the BiKEs to CD16A-mFc was tested by SPR analysis on a Biacore × 100 (GE Healthcare) using a single-cycle approach according to the manufacturer’s instructions. Briefly, pure CD16A-mFc buffered in sodium acetate (pH 5.0) was directly immobilized onto a CM5 sensor chip by the standard amine-coupling method. The reference cell was injected with *N*-hydroxysuccinimide/1-ethyl-3-(3 dimethyaminopropy) carbodiimide and ethanolamine without injection of CD16A-mFc. The BiKEs were diluted with running buffer HBS-EP (100 mM HEPES, pH 7.4, 1.5 M NaCl, 30 mM EDTA, 0.5% surfactant 20). Both mbk6 and mbk11 were assayed at concentrations of 100, 20, 4, 0.8 and 0.16 nM. The chip was regenerated with 10 mM glycine, pH 2.5, and 1 M NaCl. The sensorgram was analyzed with BiaEvaluation software, and the kinetics constants were obtained by fitting of the sensorgram.

### Enrichment of NK cells from PBMC and flow cytometry (FACS)

PBMCs were isolated from peripheral blood of healthy donors by centrifugation on a Ficoll/Hypaque gradient (GE health). NK cells were enriched from human PBMC by using the NK cell isolation kit II in the negative selection modes (Miltenyi Biotec). Purified NK cells were detected by incubation with biotinylated anti-CD56 antibody (m909, developed by our group^42^) followed by streptavidin-FITC and PE conjugated mouse anti-human CD16 antibody. Binding to NK cells was measured by incubating 10^6^ NK cells in 200 μL PBS containing 0.1% bovine serum albumin (BSA) (PBSA) with ~10 nM of BiKEs for 30 min at room temperature. The cells were washed twice with 200 μL PBSA, followed by incubation with PE-conjugated-mouse anti-FLAG antibody (Miltenyi Biotec) for 30 min on ice. After washing, the cells were used for fluorescence-activated cell sorter (FACS) analysis. The second antibody alone was used as a negative control. Binding to 293 T cells and CHO-ZA cells with or without overexpressing gp160_SC_ by these BiKEs was performed by similar procedures. These experiments were repeated three times.

### Activation of CD16A expressing Jurkat T cells

The commercially available ADCC reporter bioassay from Promega was used to evaluate the activity of mbk6 and mbk11. The Jurkat T-NFAT-Luc2-CD16A cells (Jurkat T-CD16A cell) encoding the luciferase reporter gene driven by the nuclear factor of activated T cells response element (NFAT-RE) were used as effector cells. We used a 293T-based cell line stably expressing a clade-B HIV-1 Env (gp160) (293T-gp160_SC_ cells) as target cells. Target cells were plated on 96-well plates at a density of 2.5 × 10^4^ cells in 25 μL RPMI1640 complete medium per well. 50 μL of BiKEs or mD1.22-Fc 5-fold serially diluted from initial concentration of 50 nM was added into each well. Then the effector cells were added at a density of 1.5 × 10^5^ cells in 25 ul RPMI1640 complete medium per well for a target: effector cell ratio of 1: 6. The assay was developed after 6-h or overnight incubation at 37^o^C by using the Promega Bio-Glo Luciferase Assay System (Cat. No.: G7941) according to the manufacturer’s instructions. BiKEs incubation with Jurkat T-CD16A cells in the absence of target cells or in the presence of gp160-negative 293 T cells was used to determine the non-specific activation by BiKEs serving also as negative controls in this experiment.

### IL-2 secretion

Jurkat T-CD16A cells were used to evaluate the engagement of CD16A on the effector cell surface by the BiKEs through monitoring of the IL-2 secretion. Jurkat T-CD16A cells were cultured in RPMI1640 medium (Invitrogen) containing 10% FBS, 1 mM pyruvate, 0.1 mM MEM non-essential amino acid, 250 μg/mL G418 and 100 μg/mL hygromycin (Invitrogen). Before incubation with BiKEs, Jurkat T-CD16A cells were activated overnight by Phorbol-12-myristate-13-acetate (PMA) (50 ng/ml, Sigma). Then 10^6^ Jurkat T-CD16A cells were incubated with BiKEs (20 nM) alone or with BiKEs/mouse anti-Flag mAb (1: 1000 dilution) mixture or with BiKEs opsonized target cells, 293T-gp160_SC_ cells. The mouse anti-CD16A IgG1, 3G8, was used as a positive control. After incubation, cells were centrifuged for 10 min at 1200 × g and the quantity of human IL-2 in the supernatant was measured by sandwich ELISA with the Duoset human IL-2 kit (Duoset Human IL-2, R&D Systems) according to the manufacturer’s instructions.

### NK cell degranulation

NK cell degranulation was monitored by CD107a expression after stimulation. The cell surface expression of CD107a was measured by FACS based methods as described previously^22^. Briefly, the target cells, CHO-ZA-gp160_SC_ cells or the negative control cells, CHO-ZA were plated on 96-well plates at a density of 2.5 × 10^4^ cells in 25 μL RPMI1640 complete medium per well. 50 μL of BiKEs or mD1.22-Fc were added into each well at 20 nM final concentration. The mixtures were incubated at 37^o^C for 30 min to allow for opsonization prior to addition of effector cells. Then NK cells were added at a density of 1.5 × 10^5^ cells in 25 ul RPMI1640 complete medium per well to make a target: effector cell ratio of 1: 6. PMA (1 μg/ml) and ionomycin (0.2 μg/ml) (Sigma) were used as a positive control. Thereafter, PE-Cy^TM^5 mouse anti-human CD107a (2 µl/100 µl) (Biolegend) was added for CD107a surface staining. After 1 h incubation, GolgiStop monensin (1000×, Biolegend) was added into every well and continued to incubate for 5 h. The cells were collected and washed once by FACS buffer (PBS + 0.1% BSA) and subjected to FACS analysis. BD FACSCalibur™ flow cytometer and CellQuest Pro software were used for data acquisition and analysis. NK cells were gated by forward/side scatter. The fluorescence cut-off was set so the frequency of CD107a expressing NK cells in the “NK cell only” group without adding any BiKEs and cells to be around 0.1%. This cutoff corresponds to a boundary of three standard deviations (SDs) if the dot plots obey the Gaussian distribution.

### Killing of Env-transfected and HIV-1-infected cells

Killing of Env-transfected cells was measured by a FACS-based method as described previously^43^. Briefly, CHO-ZA-gp160_SC_ cells were labelled by PKH26 (Sigma-Aldrich) according to the manufacturer’s instructions before seeding into 96-well plates at a density of 2.5 × 10^4^ cells in a 25 μL RPMI1640 complete medium per well. 50 μL of BiKEs or mD1.22-Fc with 5-fold serially diluted from 250 nM were added into each well. The mixtures of target cells and BiKEs were incubated at 37^o^C for 30 min to allow for opsonization prior to addition of effector cells. Then NK cells cultured in RPMI160 complete medium containing 200 U/ml of human recombinant IL-2 and 10 ng/ml of IL-15 (R&D Systems) were added at a density of 1.5 × 10^5^ cells in 25 ul RPMI1640 complete medium per well to make a target: effector cell ratio of 1: 6. The plates were incubated at 37^o^C for 36 hours. Then 1 μg/ml of propidium iodide (PI, Sigma) was added into wells and incubated at room temperature for 30 min. Samples were then diluted with 1:1 FACS buffer and analyzed by BD FACSCalibur™ flow cytometry. All cytotoxicity assay wells were in triplicate. The cell killing percentage was calculated by subtracting the negative controls (NK cells incubation with CHO-ZA-gp160_SC_ in the absence of BiKEs).

Killing of the chronically infected 8E5 cells by PBMCs mediated by BiKEs was evaluated by using a different assay based on measuring membrane integrity. 8E5 cells (ATCC) were plated on round-bottom 96-well plates at a density of 2.5 × 10^4^ cells in 25 μL RPMI1640 complete medium containing 20 IU/mL hIL-2 per well. 50 μL of BiKEs or mD1.22-Fc with 5-fold serially dilution from 100 nM were added into each well. The mixtures of target cells and BiKEs were incubated at 37 °C for 30 min to allow for opsonization prior to addition of effector cells. Then PBMCs were added at a density of 2.5 × 10^5^ cells in 25 ul RPMI1640 complete medium per well to make a target: effector cell ratio of 1: 10. The assay was developed after overnight incubation at 37 °C by using the Promega CytoTox-ONE™ Homogeneous Membrane Integrity Assay (Cat. No: G7890) according to the manufacturer’s instructions. IgG1 m336, a MERS-CoV-specific mAb was used as a negative control. The experiments were performed in duplicated wells. Maximum signal (Max) was achieved by treatment of the target cells with Triton X-100; target cells alone (T.C), effector cells alone and target cells with effector cells in the absence of BiKEs (no BiKE) were used as negative controls. Flurescence was measured at 560/590 nm. Cell killing percentage was calculated as (with BiKE - no BiKE)/(Max-T.C).

## Electronic supplementary material

Supplementary information
