# Supplementary material for: One-domain CD4 Fused to Human Anti-CD16 Antibody Domain Mediates Effective Killing of HIV-1-Infected Cells
Source: Sci Rep. 2017 Aug 22;7:9130. doi: 10.1038/s41598-017-07966-3 (PMC5567353; doi:10.1038/s41598-017-07966-3)
Supplement: Supplementary file 1 — Supplementary information [file 41598_2017_7966_MOESM1_ESM.pdf]

One-domain CD4 Fused to Human Anti-CD16 Antibody Domain Mediates Effective Killing of HIV-1-Infected Cells

Wei Li[1*], Yanling Wu[2], Desheng Kong[3], Hongjia Yang[4], Yanping Wang[1], Jiping Shao[1,5], Yang Feng[1], Weizao Chen[1], Liying Ma[3], Tianlei Ying[2], Dimiter S. Dimitrov[1]*

Supplemental Methods

Comparison of gp160 expression levels on 293T and CHO-ZA cell surface. The expression levels of gp160sc on 293T and CHO-ZA cells after transfection were compared by FACS. The CD4 binding site mAbs, b12 and LSEVh-LS-F[1] (one-domain CD4 fusion protein produced in our laboratory) was used for binding to cell surface associated gp160. Phycoerythrin (PE) conjugated goat anti-human IgG (γ-chain specific) (Cat. No. P9170) was used as detection antibody. FACS experimental procedure was similar as that described in the "methods" section.

Intracellular IFNγ staining. Cells were processed similarly as for the CD107a staining. However, unlike in the case of CD107a detection, the APC mouse anti-human IFNγ (Biolegend) was added after 6 h incubation and after cells fixation and permeabilization. Specifically, after adding the cells and BiKEs followed by 1 h incubation, brefeldin (1000×, Biolegend) was added into the wells and kept incubation at 37 °C for additional 5 h. Cells were collected and washed twice by PBSA. Cells were then fixed and permeabilized by using FIX&PERM® Cell Fixation&Cell Permeabilization Kit (Invitrogen) according to the manufacturer's instructions. Then cells were stained by 2 ul of APC-anti-human-IFNγ followed by washing using FACS buffer, and then subjected onto FACS analysis by using FL4 channel. Data were processed in a similar way as the CD107a staining experiments.

Killing of gp160-transfected cells competed by D6. Killing of Env-transfected cells was measured by a FACS-based method as described above and previously (22). The major difference was that D6 was added at 10 nM final concentration to the mixtures of target cells and

BiKEs when they were incubated at 37 ^{o}C for 30 min to allow for opsonization prior to addition of effector cells.

Killing of HIV-1 infected CEM cells by PBMCs mediated by BiKEs. CEM.$NK^{R}CCR5^{+}$ cells were infected with HIV-$1_{NL4\text{-}3}$ (T cell tropic X4 virus) by a spinoculation method. Four days post-infection, Env expression was detected by LSEVh-LS-F staining. In the ADCC assays the cell killing was detected by monitoring LDH release using Promega CytoTox-ONE™ Homogeneous Membrane Integrity kit. The assay was performed in round-bottom, tissue culture-treated polystyrene 96-well plates. Prior to the assay, infected cells or uninfected cells (negative controls) were washed, and seeded with a density of 2.5×10^4 cells in 25 μL RPMI1640 complete medium containing 20 IU/mL IL-2 per well. Then ~0.8 nM of BiKEs or mD1.22-Fc (50 μL) were added followed by addition of 5×10^5 effector cells (PBMCs) to achieve a E: T ratio of 20: 1 in a 100-μl final volume. The assay plates were incubated overnight at 37°C and in 5% CO_2. The non-specific lysis due to HIV-1 infection itself was defined as 100 × (target cell control – no cell control)/(high release control – no cell control) while the BiKEs mediated lysis percentage was calculated as 100 × (with BiKE – no BiKE)/(high release control – target cell control), where target cell control refers to the spontaneous LDH release by HIV-1 infected CEM cell alone; no cell control is the R10 culture medium alone; high release control is the high lysis of target cells by 1% Triton X-100; no BiKE means that the LDH release of PBMCs + infected CEM cells and with BiKE is when BiKE is added.

Supplemental Figure Legends

Supplemental Figure 1. comparison of gp160 expression levels on 293T and CHO-ZA cells after transfection. Gp160sc expression was FACS detected using b12 or one-domain CD4 fusion protein followed by PE conjugated goat anti-human IgG. **(a)** b12 binding to 293T-gp160sc. Black, pink and blue lines represent the blank 293T-gp160sc cells, cells incubation with second antibody only and cells incubation with b12 followed by second antibody. **(b)** b12 binding to CHO-ZA-gp160sc. Black, pink and orange lines represent the blank CHO-ZA-gp160sc cells, cells incubation with second antibody only and cell incubation with b12 followed by second antibody. Experiments were performed in triplicate. **(c)** and **(d)** Corresponding binding of LSEVh-LS-F to 293T cells and CHO-ZA-gp160sc cells, respectively.

Supplemental Figure 2. IFNγ expression of NK cells. NK cells alone or incubated with BiKEs alone were used as negative controls, and those stimulated by PMA/Ionomycin as a positive control. Cells were fixed followed by permeabilized before IFNγ staining. Intracellular IFNγ staining by the APC mouse anti-human IFNγ antibody was detected by FACS. **(a)** Flow cytometry of NK cells. **(b)** Quantitation and statistical evaluation of the flow cytometry data. Data was analyzed in similar ways as for the CD107a staining. Significant differences were analyzed by Student's t test. A p value < 0.05 was considered significant. *: $p < 0.05$. **: $p < 0.01$. ***: $p < 0.001$. NS: not significant.

Supplemental Figure 3. mbk6 mediated target cell killing in the presence of D6. Experiments were performed as those shown in Fig. 6 except the serially diluted mbk6 proteins contained 10 nM of D6. Experiments were performed in triplicate.

Supplemental Figure 4. PBMCs killing of CEM.NKR.CCR5 cells infected with HIV-1_{NL4-3}. (**a**) Schematic diagram of the experimental design. (**b**) FACS detection of Env-expression in infected CEM cells by LSEVh-LS-F staining. (**c**) The percentage of killed HIV-1 infected CEM cells monitored by the LDH release after co-incubation with hPBMCs as effector cells in the presence of 0.8 nM of BiKEs or mD1.22-Fc.

Supplemental Figure 1

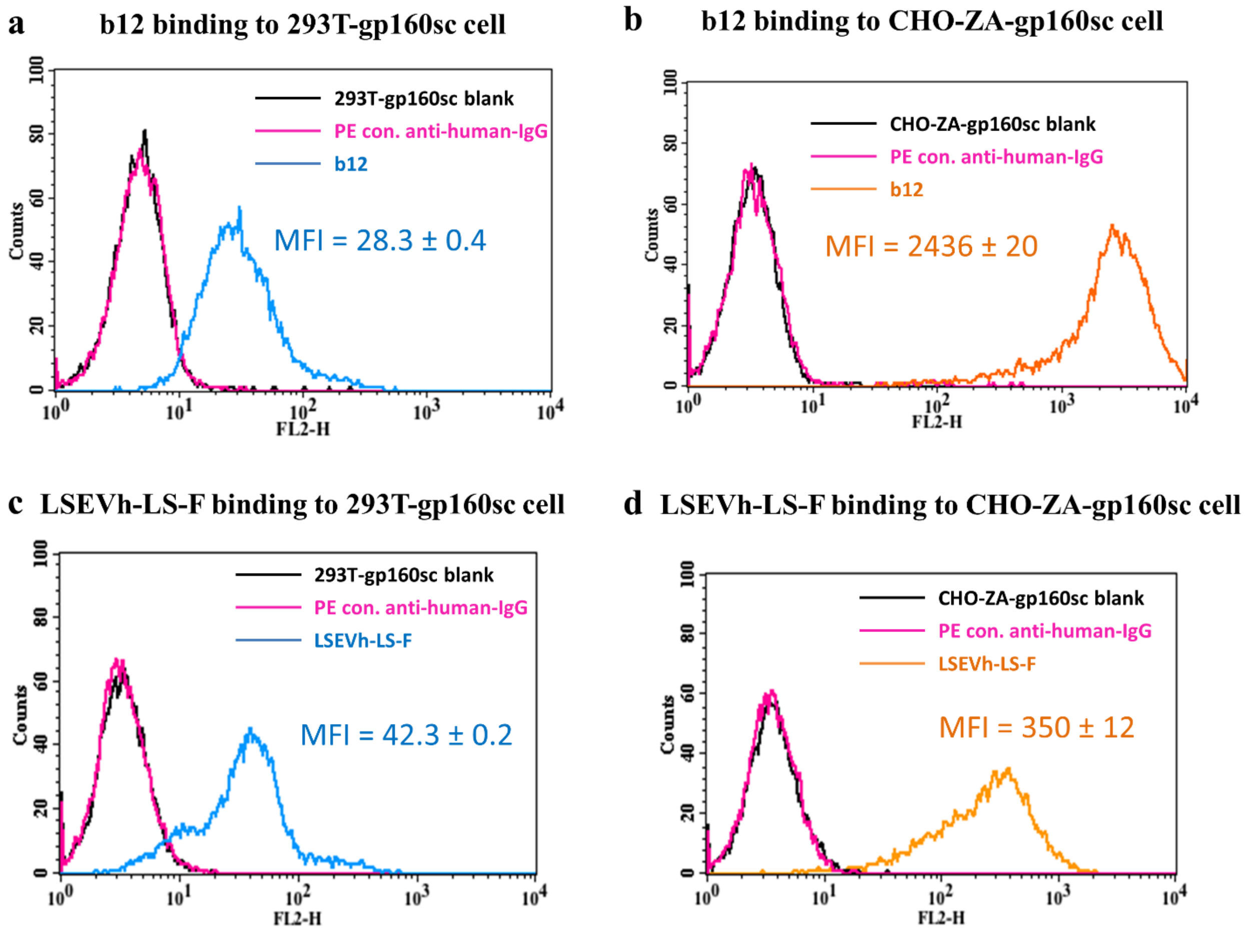

Supplemental Figure 2

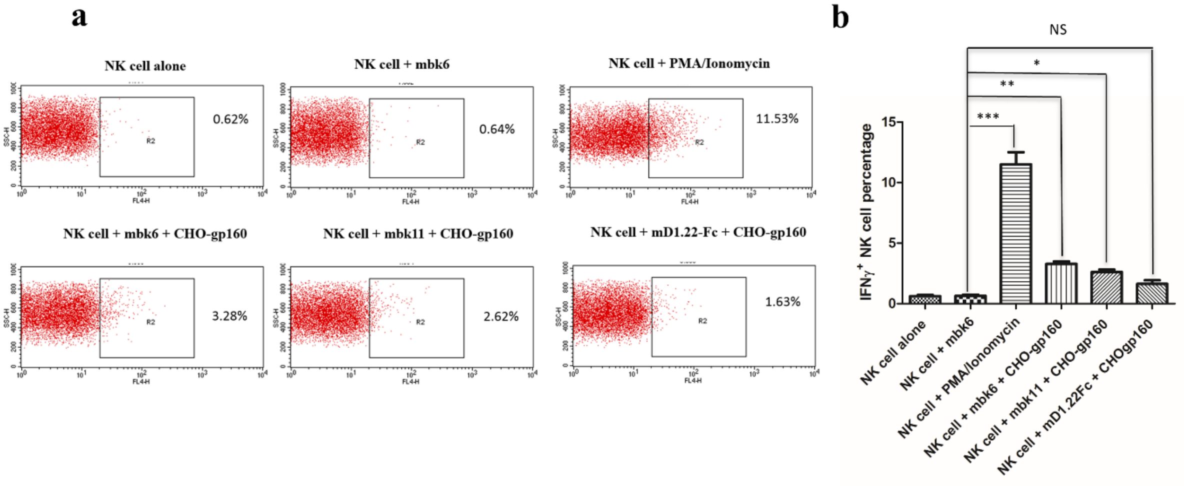

Supplemental Figure 3

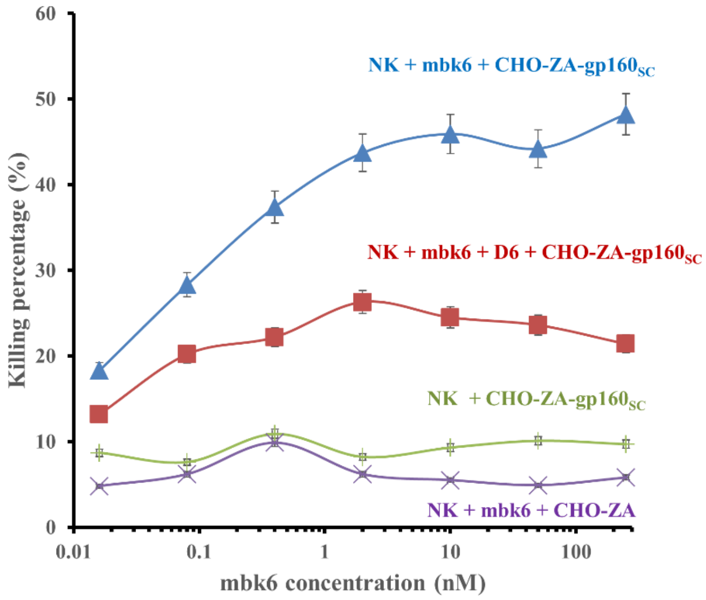

Supplemental Figure 4

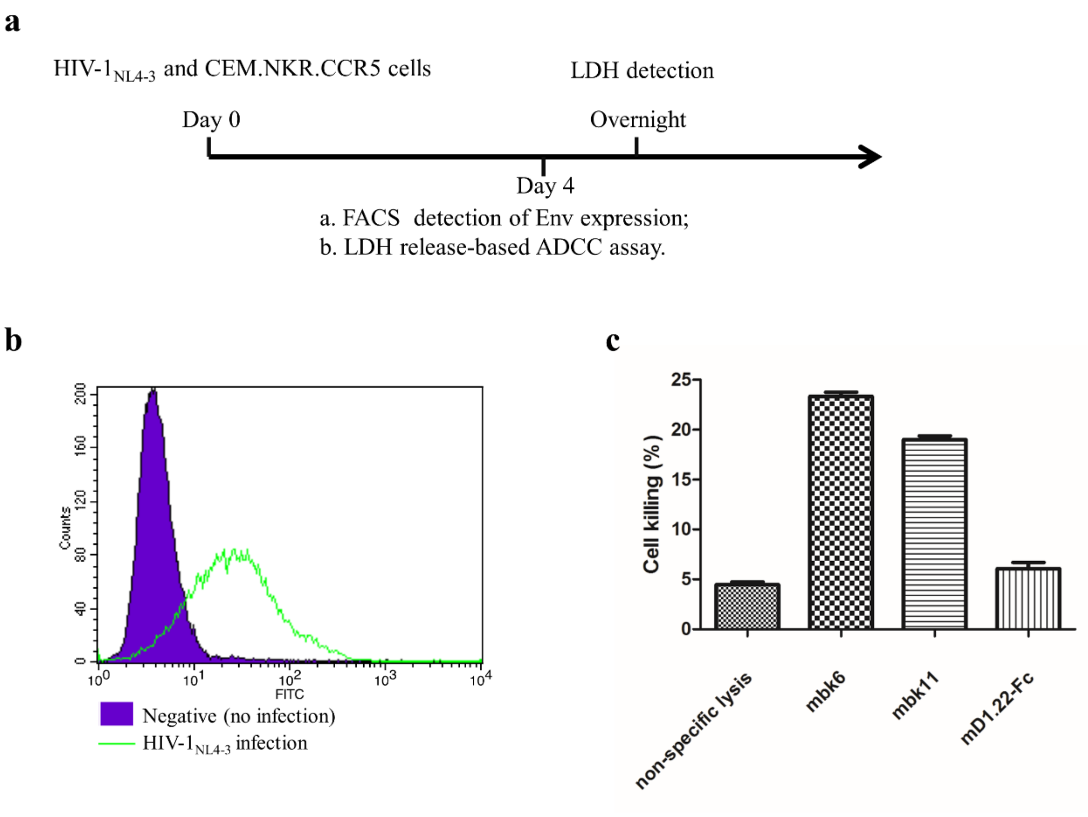

1 Chen, W. *et al.* Improving the CH1-CK heterodimerization and pharmacokinetics of 4Dm2m, a novel potent CD4-antibody fusion protein against HIV-1. *mAbs* **8**, 761-774, doi:10.1080/19420862.2016.1160180 (2016).
